# Low Vitamin D and C Levels are Associated with Increased Disease Activity in Jordanian RA and SLE Patients: A Case-Control Study

**DOI:** 10.2174/0118715303424335251128113015

**Published:** 2026-01-16

**Authors:** Faten S. Tout, Baraah Aljaafreh, Nawal Hijjawi, Omar Abuyaman, Baraah Azaizeh

**Affiliations:** 1 Department of Medical Laboratory Sciences, Faculty of Applied Medical Sciences, The Hashemite University, Zarqa 13133, Jordan;; 2 Department of Laboratory Medical Sciences, Mutah University, Al-Karak, Jordan

**Keywords:** Rheumatoid arthritis, vitamin D, inflammation, vitamin C, autoimmunity, dietary supplements

## Abstract

**Introduction:**

Rheumatoid arthritis (RA) and systemic lupus erythematosus (SLE) are chronic autoimmune diseases characterized by inflammation and tissue damage. Emerging evidence suggests vitamins D, C, and K may influence disease activity. The aim of this study is to assess serum vitamin levels in Jordanian RA and SLE patients and their association with disease activity.

**Methods:**

This case-control study included 156 participants recruited at the University of Jordan Hospital (January-September 2023): 99 patients with RA, 11 patients with SLE, and 46 healthy controls. Serum vitamin levels were measured by ELISA; inflammatory markers were extracted from medical records. Statistical analysis was performed using SPSS with significance set at *p*<0.05.

**Results and Discussion:**

RA and SLE patients showed significantly lower vitamin D (RA: 17.5 ±16.4 ng/ml, adjusted *p*=0.003; SLE: 13.7 ±8.3 ng/ml, adjusted *p*=0.044) and vitamin C (RA: 14.7 ± 5.3 mg/ml, adjusted *p*=0.003; SLE: 13.5 ± 3.5 ng/ml, adjusted *p*=0.003) levels versus controls (D: 33.3 ±20.9 ng/ml; C: 38.8 ±33.4 ng/ml). Vitamin K levels were reduced but remained within the reference range (RA: 2.2±1.1 ng/ml, adjusted *p*=0.003; SLE: 2.9±1.0 ng/ml, adjusted *p*=0.003). Deficiencies in vitamins D and C were found to be positively associated with elevated inflammatory markers, particularly ESR. No significant associations were found between vitamin levels and anti-CCP, ANA, joint involvement, or skin rash in RA and SLE patients; however, vitamin C levels were significantly associated with anti-CCP antibody expression in RA patients.

**Conclusion:**

In Jordanian RA and SLE patients, lower serum levels of vitamin D and C are associated with increased inflammation, underscoring the need for further research to explore the therapeutic potential of supplementation.

## 1 INTRODUCTION

Rheumatoid arthritis (RA) is a chronic systemic autoimmune disorder characterized by persistent synovial inflammation, leading to cartilage destruction, bone erosion, and joint deformity [1]. RA also affects extra-articular systems, such as cardiovascular, pulmonary, and ocular systems, significantly increasing morbidity and mortality in patients with RA [2]. Clinically, RA typically presents with symmetrical polyarthritis, prolonged morning stiffness, and elevated inflammatory markers [3]. RA is more common in women (female-to-male ratio of ~ 3:1), and its pathogenesis involves a complex interplay of genetic susceptibility and environmental triggers, such as smoking and infection [4]. Diagnosis relies on clinical assessments, imaging, and serological markers (*e.g.*, Rheumatoid factor (RF) and anti-citrullinated protein antibodies (ACPAs)) [5]. Treatment strategies usually focus on the use of disease-modifying anti-rheumatic drugs (DMARDs) to slow disease progression alongside non-pharmacologic interventions, such as physical therapy and lifestyle modifications [5].

In contrast, systemic lupus erythematosus (SLE) is a multisystem autoimmune disease characterised by dysregulation of the immune response, particularly the production of autoantibodies against nuclear antigens, leading to widespread inflammation and tissue damage [6]. SLE can affect any organ system, with common manifestations, including cutaneous rashes, arthritis, nephritis, and neuropsychiatric symptoms [7]. SLE predominantly affects females, particularly women of childbearing age [8]. The aetiology involves genetic predispositions and environmental factors, such as ultraviolet light exposure and viral infections [9, 10]. Diagnosis of SLE is based on a combination of clinical features and serological markers, such as antinuclear antibodies (ANA) and anti-double-stranded DNA (dsDNA) antibodies [6]. SLE management involves a combination of glucocorticoids, immunosuppressants, and biologic agents tailored to disease severity and organ involvement [6].

Vitamins are essential for normal physiological functions, immune homeostasis, and modulation of inflammation [11]. Vitamin D (VD) has immunomodulatory effects by regulating the differentiation and function of T cells, B cells, and dendritic cells [12]. Vitamin C (VC), a potent antioxidant, is crucial for collagen synthesis and the neutralisation of reactive oxygen species (ROS), which are elevated in autoimmune conditions and contribute to tissue damage [13]. Vitamin K (VK) regulates calcium homeostasis and inhibits vascular calcification, processes often dysregulated in chronic inflammatory diseases [14]. Emerging evidence indicates that immune cells, including monocytes, dendritic cells, and activated T cells, express receptors for these vitamins, underscoring their regulatory roles in diverse immune functions. Low levels of these vitamins have been linked to increased disease activity in autoimmune conditions, such as RA and SLE, suggesting they may influence disease progression [11, 15].

Despite growing evidence linking vitamin deficiencies to autoimmune diseases, data on the prevalence and clinical significance of vitamins D, C, and K deficiencies in patients with RA and SLE, especially in Jordan, remain limited. Although Jordan’s geographic location suggests adequate sunlight for VD, lifestyle, dietary habits, and religious practices (*e.g.*, clothing choices) may contribute to suboptimal vitamin levels [16]. Furthermore, the association between these vitamins and disease activity in RA and SLE is underexplored in this region. This study aims to evaluate serum levels of vitamins D, C, and K in Jordanian patients with RA and SLE and investigate their relationship with disease severity and activity. The findings may inform potential therapeutic and nutritional interventions to improve patient outcomes.

## 2 MATERIALS AND METHODS

### 2.1 Ethical Approval, Selection Criteria, Data Collection, and Sample Size Calculation

This case-control study was conducted between January 2023 and September 2023, adhering to the ethical guidelines established by the Institutional Review Board (IRB) of Hashemite University (No. 2200811) and the 1964 Declaration of Helsinki and its later amendments. Ethical approval was obtained prior to the initiation of the study, and authorization was secured from the Jordanian Ministry of Health for sample collection from hospitals under its jurisdiction (No. 5146/2024). All participants provided written informed consent prior to their inclusion in the study.

Participants were recruited consecutively from Jordanian patients attending the rheumatology clinics at the University of Jordan Hospital during the study period. The study included patients with a long-standing history of RA and SLE, diagnosed by experienced rheumatologists using the 2010 American College of Rheumatology (ACR)/European League Against Rheumatism (EULAR) classification criteria for RA or the 2012 ACR/EULAR classification criteria for SLE.

Inclusion criteria required participants to have no coexisting diseases, a positive Rheumatoid factor (RF) test for RA patients, and a positive antinuclear antibody (ANA) test for SLE patients. Exclusion criteria included current vitamin supplement use, active disease flare, or incomplete data. Demographic and clinical data, such as age, gender, duration of diagnosis, and medications, were collected to identify potential confounders.

The sample size of RA patients (n=99) was calculated to achieve a power of 80% (β=0.20) at a significance level of α=0.05, based on expected differences in vitamin levels from prior studies [17, 18]. Due to the lower prevalence of SLE and recruitment challenges, only 11 SLE patients were included, and findings for this group should be interpreted as exploratory.

Serum vitamin levels were measured using Enzyme-linked immunosorbent assays (ELISA), and inflammatory markers (CRP, ESR) were extracted from medical records corresponding to the same visit. Anti-cyclic citrullinated peptide (anti-CCP) results were also retrieved from medical records. Potential confounders identified in demographic and clinical data were controlled for using multivariate statistical analyses. Missing data were minimal (<5%); cases with missing critical variables were excluded from respective analyses. No imputation methods were used.

### 2.2 Procedure

Blood samples were collected from patients *via* venipuncture following standard protocols, using 5 ml venous blood in serum separation tubes (SST) [19]. To ensure patient confidentiality, each sample was assigned a unique identification number without any personal identifiers. After collecting, SST samples were allowed to clot for 30 minutes, then centrifuged at 3500 rpm for 5 minutes to separate the serum. The resulting serum was aliquoted into four cryovials and stored at -50°C for subsequent analysis.

### 2.3 ELISA

ELISA assays were performed according to the manufacturer's instructions to quantify the levels of vitamins D, C, and K in the samples. The kits used were VD (Kit No. 9425-300; Monobind, USA), VC (Kit No. 20231011; Sunlong Biotech, China), and VK (Kit No. 20231012; Sunlong Biotech). Absorbance readings were obtained using a BioTek ELISA plate reader (USA) at the specified wavelengths for each vitamin. Calibration curves were generated using Microsoft Excel, and the concentrations of vitamins D, C, and K in each sample were calculated by interpolating the absorbance values against these calibration curves.

### 2.4 Statistical Analysis

Data collection and sorting were conducted using Microsoft Excel (supplementary information), while statistical analyses were performed using the Statistical Package for Social Sciences (IBM SPSS, version 26). A significance level of α = 0.05 was set, and *p*-values < 0.05 were considered statistically significant. Descriptive statistics (frequencies, percentages, means, and standard deviations) summarized demographic and clinical characteristics.

Normality of continuous variables was assessed using the Shapiro-Wilk test. For comparisons between independent groups (*e.g.*, vitamin levels in RA *vs*. healthy controls), independent t-tests were used for normally distributed data, and the Mann-Whitney U test was applied for non-normally distributed data or small subgroups (*e.g.*, SLE group). Categorical variables (*e.g.*, VD levels by anti-CCP status) were analyzed using Chi-squared tests for RA patients and Fisher's exact test for the smaller SLE group. To account for multiple hypothesis testing and reduce the risk of Type I errors, the Benjamini-Hochberg procedure was applied to control the false discovery rate (FDR) at 5%, and adjusted *p*-values were calculated for all multiple comparisons. *P*-values were interpreted considering both statistical significance and clinical relevance, with attention to the power limitations of smaller subgroups.

## 3 RESULTS

### 3.1 Demographic and Clinical Data

A total of 156 participants were recruited for this study and categorized into three groups based on their diagnosis: 11 with SLE, 99 with RA, and 46 healthy controls. The mean age of participants was 34.2 ± 15.9 years in the SLE group and 46.4 ± 13.6 years in the RA group. The average disease duration was 5.1 ± 3.6 years. The majority of RA patients were female (n=85, 85.9%), while all SLE patients were female (n=11, 100%) (Table **1**). The geographic distribution of RA and SLE patients revealed a significant variation across different cities in Jordan. The majority of patients (RA: 55.6% and SLE: 81.8%) were from Amman, the capital city, followed by Zarqa (30%) and Al Karak (11.1%) in RA patients (Table **1**). This distribution reflects the concentration of healthcare resources in urban centres, particularly Amman, which hosts the majority of specialised rheumatology clinics and advanced treatment facilities.

The recruited patients exhibited a wide range of clinical manifestations (Table **2**). In RA patients, large joint swelling was prevalent, with 61.6% (n=61) experiencing small joint swelling and 34.3% (n=34) exhibiting large joint swelling. In SLE patients, small joint swelling was the most common manifestation, observed in 45.5% (n=5), followed by skin rash and hair loss in 36.6% (n=4) of cases. These findings highlight the diverse clinical presentations in these autoimmune conditions, emphasising the need for tailored assessments and interventions. All RA and SLE patients received a combination of glucocorticoids (prednisone), hydroxychloroquine, methotrexate, and/or analgesics.

### 3.2 Vitamin Levels Among Healthy, SLE, and RA Groups

VD and VC deficiencies, defined as levels below 20 ng/ml and 14 ng/ml, respectively, were predominantly observed in RA and SLE patients compared to healthy controls [20]. Among RA patients, 81 of 99 exhibited VD deficiency, 53 had VC deficiency, while only 18 and 46 patients had sufficient levels of VD and VC, respectively. In SLE patients, 10 out of 11 had deficient or insufficient VD levels, with only one patient showing sufficient levels. Additionally, 7 out of 11 SLE patients displayed VC deficiency.

Quantitative analysis revealed that RA patients had significantly lower mean VD (17.5 ± 16.4 ng/ml, adjusted *p*=0.003) and VC (14.7 ± 5.3 ng/ml, adjusted *p*=0.003) levels compared to healthy controls. Similarly, SLE patients exhibited significantly lower levels of VD (13.7 ± 8.3 ng/ml, adjusted *p*=0.044) and VC (13.5 ± 3.5 ng/ml, adjusted *p*=0.003). Healthy controls had vitamin levels within the recommended normal range (33.3 ± 20.7 ng/ml for VD and 38.8 ± 33.4 ng/ml for VC) (Table **3**). These findings demonstrate an association between VD and VC deficiencies and the presence of autoimmune disease in RA and SLE patients.

Regarding VK levels, all groups fell within the reference range; however, healthy controls had the highest VK levels at 5.8±2.5 ng/ml. RA patients had significantly lower VK levels (2.2±1.1 ng/ml, adjusted *p*=0.003), as did SLE patients (2.9±1.0 ng/ml, adjusted *p*=0.003), compared to healthy controls (Table **3**). These statistically significant differences highlight a relative VK deficiency in RA and SLE patients, even when values remain within clinically accepted limits, underscoring the importance of nutritional monitoring in these populations.

### 3.3 The Influence of Suboptimal and Deficient Vitamin Levels on RA and SLE Disease Activity

We examined the relationship between CRP and ESR levels and VD and VC levels in patients with RA and SLE. CRP and ESR are established inflammatory markers that increase due to chronic inflammation, making their monitoring essential for assessing disease activity in autoimmune conditions [21].

Our analysis showed a significant elevation in ESR among RA patients with decreased levels of VD (52.8 ± 33.1 mm/hr, adjusted *p*=0.016) and VC (54.1 ± 32.7 mm/hr, adjusted *p*=0.04), indicating an association between vitamin deficiencies and increased inflammation in this group. Although CRP levels were slightly elevated in RA patients with insufficient VD, this increase was not statistically significant (Table **4**).

In SLE patients, low VD levels were associated with a 1.9-fold increase in CRP compared to those with sufficient VD, but no significant correlation was found between low VD levels and ESR. VC deficiency was not associated with ESR or CRP in SLE patients (Table **4**).

Overall, lower levels of VD and VC are associated with elevated inflammatory markers, particularly ESR, in RA, while this relationship is less pronounced in SLE.

We further investigated the relationships among VD and VC levels, anti-CCP status, and disease progression, given the importance of anti-CCP in predicting disease progression [22]. Among RA patients, most did not express anti-CCP antibodies (n=68), and no significant association was found between VD levels and anti-CCP expression. In SLE patients, there was also no significant association between VD levels and anti-CCP antibodies, despite a majority (n=8) testing positive (Table **5**).

VC levels were significantly associated with anti-CCP antibody expression in RA patients but not in SLE patients (Table **5**). This suggests that VC deficiency may increase the likelihood of anti-CCP antibody expression in RA, indicating a potential role for VC in modulating the autoimmune response in this condition.

We further explored the relationships between VD and VC levels and the expression of clinical signs and symptoms in RA and SLE patients, as assessed by the disease activity score (DAS-28) and the systemic lupus erythematosus disease activity index (SLEDAI), respectively. These disease activity indices evaluate manifestations, such as joint swelling in RA and skin rash in SLE, along with other clinical features. However, we found no significant associations between insufficient or deficient vitamin levels and these clinical signs in either patient group (Table **6**). These findings suggest that factors other than vitamin levels influence symptom manifestation in these autoimmune conditions, highlighting a complex interplay between vitamin status and disease presentation.

In conclusion, RA and SLE patients showed significant deficiencies in VD and VC compared to healthy individuals. In RA patients, VD and VC deficiencies were associated with elevated ESR, suggesting a link between vitamin status and disease activity. While findings for SLE patients were exploratory due to a limited sample size, these results highlight the potential role of vitamin supplementation in managing autoimmune conditions, particularly in RA.

## 4 DISCUSSION

VD plays a critical role in calcium metabolism, cellular growth, differentiation, and immune regulation [23]. Its involvement in RA and SLE has been widely studied, though findings vary due to geographic, ethnic, and methodological differences [24-26]. VC, a key antioxidant, supports immune function and reduces oxidative stress, which is elevated in autoimmune conditions like RA and SLE [27, 28]. VK, traditionally linked to coagulation, may also exhibit anti-inflammatory effects relevant to these conditions [29, 30].

This study identified a high prevalence of VD and VC deficiencies in RA and SLE patients, particularly among RA cases. While VK levels remained within the normal range, they were significantly lower in patients than in healthy controls. These findings are consistent with previous studies demonstrating reduced vitamin levels in patients with autoimmune diseases [25-27, 31].

Factors contributing to these deficiencies include joint pain and fatigue limiting mobility, sun exposure, and dietary intake [32]. Notably, mobility issues were more pronounced among urban patients, particularly those from Amman, suggesting better healthcare access. In contrast, fewer patients from smaller cities like Karak faced potential barriers to healthcare access, including limited resources and financial constraints for travelling to specialised centres. This aligns with literature highlighting urban-rural disparities in rheumatic disease management, where socioeconomic status and geographic location significantly influence health outcomes [17, 18]. Additionally, medications, such as glucocorticoids, can disrupt the metabolism of VD and VC, potentially contributing to the observed deficiencies in our study. Notably, all patients in this study were receiving glucocorticoid therapy, which may have influenced both vitamin levels and inflammation markers [33, 34]. Renal complications in SLE patients can hinder VD activation, while gastrointestinal issues may interfere with the absorption and metabolism of VC and VK [35-37]. Other individual factors, such as body mass index (BMI), age, genetics, and dietary habits, also contribute to deficiencies in these vitamins [38]. The chronic nature of RA and SLE exacerbates oxidative stress and tissue damage, increasing the utilisation of antioxidants like VC and leading to their depletion [39].

Likewise, the reduced levels of VK observed in patients with RA and SLE, compared to healthy controls, may indicate its consumption in managing inflammation. A study indicated that VK possesses anti-inflammatory properties that can prevent joint degradation by inhibiting the NF-κB signalling pathway and reducing MMP-3 production, an enzyme involved in collagen degradation [40]. Alterations in vitamin K-dependent proteins, such as GAS6 and protein S, have been associated with SLE disease activity, underscoring the importance of VK in immune regulation and inflammation management in autoimmune diseases [41].

We observed an association between VD and VC deficiencies and increased inflammation markers (CRP, ESR), suggesting these vitamins may influence disease activity. VD effect is likely associated with its capacity to inhibit pro-inflammatory cytokine production by interacting with the vitamin D receptor (VDR) on immune cells, mediated by NF-κB and STAT3 signalling pathways [42]. Similarly, studies indicate an inverse relationship between VC levels and CRP, supporting its anti-inflammatory properties [43]. Supplementation can reduce CRP levels, particularly in younger, non-smoking individuals at doses below 500 mg/day [43]. Additionally, VC reduces biomarkers of oxidative stress, such as F2-isoprostanes, which correlate with CRP levels [44]. Furthermore, anti-CCP antibodies in RA increase oxidative activity in synovial fluid, indicated by higher levels of malondialdehyde (MDA) and myeloperoxidase (MPO), potentially contributing to RA severity and the observed association with VC status [39, 45]. Persistent inflammation drives RA and SLE symptoms, as assessed by DAS-28 and SLEDAI, respectively, with joint swelling and skin manifestations. Our study found no significant association between VD or VC deficiency and clinical signs, potentially due to the small SLE sample size, genetic variability, or age differences. This is consistent with mixed results reported in prior work [46-48]. Despite promising data linking vitamin levels to inflammation, supplementation remains a preliminary intervention requiring further randomized controlled trials to confirm efficacy and safe dosing.

## CONCLUSION

Our findings highlight the prevalence of vitamin deficiencies in RA and SLE and their relationship with inflammation, supporting the potential utility of routine vitamin level assessments as part of disease monitoring. While supplementation shows promise for reducing inflammation, definitive therapeutic recommendations await confirmation from larger, well-designed clinical trials.

The strengths of this study include the concurrent examination of multiple vitamins (D, C, and K) in RA and SLE patients, the application of rigorous statistical corrections, such as the Benjamini-Hochberg procedure to control the false discovery rate, and the integration of clinical, biochemical, and antibody status data for comprehensive analysis.

## STUDY LIMITATIONS

Limitations include a small, statistically imbalanced SLE cohort, which limits generalizability and power to detect associations, thereby increasing the risk of Type II error. Single-season sampling may affect vitamin levels due to seasonal variation, and potential selection and measurement biases inherent in the study design must be considered. Further research should incorporate larger, balanced cohorts with longitudinal designs and detailed confounder assessment, including diet, BMI, socioeconomic factors, and seasonal variation, to elucidate causality and refine supplementation strategies.

## Figures and Tables

**Table 1:** Demographic and geographic characteristics of patients with Rheumatoid arthritis (RA), systemic lupus erythematosus (SLE) and healthy controls.

General Characteristics		Frequency n (%)		
		RA (99)	SLE (11)	Control (46)
Age	Mean ± SD	46.4 ± 13.6	34.8 ± 15.9	37.8 ± 10.5
Gender	Female	85 (85.9)	11 (100)	37 (80.4)
	Male	14 (14.1)	0 (0.0)	9 (19.6)
Geographic distribution	Amman	55 (55.6)	9 (81.8)	25 (54.3)
	Zarqa	30 (30.2)	-	9 (19.7)
	Kark	11 (11.1)	-	12 (26.0)
	Other	3 (3.1)	2 (18.2)	-

**Abbreviations:** N: Number, %: Percentage, SD: Standard Deviation. RA: Rheumatoid Arthritis; SLE: Systemic Lupus Erythematosus.

**Table 2:** Frequency of clinical manifestations including joint inflammation, skin rash and hair loss in patient with Rheumatoid arthritis (RA) and systemic lupus erythematosus (SLE).

Clinical Manifestations			Frequency N (%)	
			RA	SLE
Joint Inflammation	Small joint swelling	Present	61 (61.6)	5 (45.5)
		Absent	38 (38.4)	6 (54.6)
	Intermediate joint swelling	Present	2 (2.0)	1 (9.1)
		Absent	97 (98.0)	10 (90.9)
	Large joint swelling	Present	34 (34.3)	1 (9.1)
		Absent	65 (65.7)	10 (90.9)
Skin rash		Present	-	5 (45.6)
		Absent		6 (54.5)
Hair loss		Present		4 (36.4)
		Absent		7 (63.6)

**Abbreviations:** N: Number, %: Percentage. RA: Rheumatoid Arthritis; SLE: Systemic Lupus Erythematosus.

**Table 3:** Comparison of serum vitamin d, vitamin c, and vitamin k levels among Rheumatoid arthritis (RA), systemic lupus erythematosus (SLE), and healthy controls.

Vitamin D (ng/ml)	Study group	Mean ± SD	*p*-Value^1^	Adjusted *p*-Value	Significant (FDR 5%)	CI (95%)
	**Healthy control**	33.3 ± 20.9	**-**	**-**	**-**
	**RA**	17.5 ± 16.4	<0.001*	0.003*	Yes	[14.3, 20.8]
	**SLE**	13.7 ± 8.3	0.029*	0.044*	Yes	[8.1, 19.3]
**Vitamin C (ng/ml)**	**Healthy control**	38.8 ± 33.4	**-**	**-**	-	-
	**RA**	14.7 ± 5.3	<0.001*	0.003*	Yes	[13.7, 15.8]
	**SLE**	13.5 ± 3.5	<0.001*	0.003*	Yes	[11.2, 15.9]
**Vitamin K** **(ng/ ml)**	**Healthy control**	5.8 ± 2.5	**-**	**-**	-	-
	**RA**	2.2 ±1.1	<0.001*	0.003*	Yes	[2.0, 2.5]
	**SLE**	2.9 ± 1.0	<0.001*	0.003*	Yes	[2.3, 3.6]

**Note:** *p*-Value^1^ were determined using independent t-test for RA patients and Mann-Whitney U for SLE. Adjusted *p*-Values were calculated using the Benjamini-Hochberg procedure to control the false discovery rate (FDR) at 5%. *Statistical significance was defined as adjusted *p* < 0.05. For small sample sizes, confidence intervals may be wide and should be interpreted with caution. **Abbreviations:** SD: Standard Deviation; CI: Confidence Interval; RA: Rheumatoid Arthritis; SLE; Systemic Lupus Erythematosus.

**Table 4:** Inflammatory markers C-reactive protein (CRP) and erythrocyte sedimentation rate (ESR) in Rheumatoid arthritis (RA), systemic lupus erythematosus (SLE) patients in relation to vitamin D And vitamin C status.

Disease	Inflammatory Markers	Vitamin D Levels (ng/ml)					Vitamin C Concentration (ng/ml)				
		Deficient and Insufficient	Sufficient	*p*-Value^1^	Adjusted *p*-Value	FDR (5%)	Deficient	Sufficient	*p*-Value^1^	Adjusted *p*-Value	FDR (5%)
RA	ESR (mm/hr.)	52.8 ± 33.1(CI: [45.6, 60.0])	34.6 ± 25.0(CI: [22.3, 46.8])	0.004*	0.016*	Yes	54.1 ± 32.7(CI: [45.3, 62.9])	39.9 ± 25.4(CI: [31.6, 48.3])	0.015*	0.040*	**Yes**
	CRP (mg/dl)	2.6 ± 3.6(CI: [1.8, 3.4])	2.1 ± 3.2(CI: [0.5, 3.6])	0.259	0.345	No	1.8 ± 1.4(CI: [1.4, 2.2])	1.8 ± 1.5(CI: [1.4, 2.3])	0.445	0.445	No
SLE	ESR (mm/hr.)	48.2 ± 27.7(CI: [28.4, 68.0])	60.00	0.100	0.133	No	38.2 ± 20.1(CI: [22.2, 54.2])	69.0 ± 26.6(CI: [28.9, 109.1])	0.050*	0.067	No
	CRP (mg/dl)	1.9 ± 1.7(CI: [0.7, 3.1])	0.00	0.005*	0.016*	Yes	01.2 ± 0.9(CI: [0.5, 1.9])	2.7 ± 2.5(CI: [0.0, 5.8])	0.900	0.900	No

**Note:** *p*-Value^1^ were determined using independent t-test for RA patients and Mann-Whitney U for SLE. Adjusted *p*-Values were calculated using the Benjamini-Hochberg procedure to control the false discovery rate (FDR) at 5%. *Statistical significance was defined as adjusted *p* < 0.05. Vitamin D levels: Deficient < 20 ng/ml, Insufficient 20-30 ng/ml, Sufficient > 30 ng/ml. Vitamin C and Vitamin K cutoff values: 14 ng/ml and 0.50 ng/ml respectively. For small sample sizes, confidence intervals may be wide and should be interpreted with caution. **Abbreviations:** SD: Standard Deviation; CI: Confidence Interval; CRP: C-Reactive Protein; ESR: Erythrocyte Sedimentation Rate; RA: Rheumatoid Arthritis; SLE: Systemic Lupus Erythematosus.

**Table 5:** Anti-cyclic citrullinated peptide antibody (Anti-CCP) status in Rheumatoid arthritis (RA), systemic lupus erythematosus (SLE) patients in relation to vitamin D and vitamin C levels.

Anti-CCP Status		Vitamin D Levels (ng/ml)		*p*-Value^1^	Adjusted *p*-Value	FDR (5%)	Vitamin C Levels (ng/ml)		*p*-Value^1^	Adjusted *p*-Value	FDR (5%)
		Deficient / Insufficient	Sufficient				Deficient	Sufficient			
RA patients	Negative	54	14	0.358	0.477	No	25	39	0.001*	0.005*	**Yes**
	Positive	27	4				23	8			
SLE patients	Negative	2	1	0.087	0.486	No	6	3	1.000	1.000	No
	Positive	8	0				1	1			

**Note:** *p*-Value^1^ was determined using independent chi-square test for RA patients and Fisher's exact test for SLE patients. Adjusted *p*-Values were calculated using the Benjamini-Hochberg procedure to control the false discovery rate (FDR) at 5%. *Statistical significance is defined was defines as *p* < 0.05. Vitamin D levels: Deficient < 20 ng/ml, Insufficient 20-30 ng/ml, Sufficient > 30 ng/ml. Vitamin C cut off values: 14 ng/ml. For small sample sizes, confidence intervals may be wide and should be interpreted with caution. **Abbreviations:** RA: Rheumatoid Arthritis; SLE: Systemic Lupus Erythematosus; anti-CCP: Anti-Cyclic Citrullinated Peptide antibody.

**Table 6:** Frequency of clinical manifestations among Rheumatoid arthritis (RA) and systemic lupus erythematosus (SLE) patients by vitamin D and vitamin C status.

Clinical Manifestations			Vitamin D (ng/ml)		*p*-Value^1^	Adjusted *p*-Value	FDR (5%)	Vitamin C (ng/ml)		*p*-Value^1^	Adjusted *p*-Value	FDR (5%)
			Deficient / Insufficient	Sufficient				Deficient	Sufficient			
RA patients	Small joint swelling	Yes	50	11	0.960	0.96	No	31	30	0.492	0.617	No
		No	31	7				22	16			
	Large joint swelling	Yes	29	5	0.516	0.617	No	21	13	0.235	0.376	No
		No	52	13				32	33			
SLE patients	Small Joint involvement	Yes	4	1	0.486	0.617	No	3	2	1.000	1.000	No
		No	6	0				4	2			
	Skin rash	Yes	4	1	0.486	0.617	No	4	1	0.617	0.617	No
		No	6	0				3	3			

**Note:** *p*-Value^1^ was determined using independent chi-square test for RA patients and Fisher's exact test for SLE patients. Adjusted *p*-Values were calculated using the Benjamini-Hochberg procedure to control the false discovery rate (FDR) at 5%. Vitamin D levels: Deficient < 20 ng/ml, Insufficient 20-30 ng/ml, Sufficient > 30 ng/ml. Vitamin C cut off values: 14 ng/ml. For small sample sizes, confidence intervals may be wide and should be interpreted with caution. **Abbreviations:** RA: Rheumatoid Arthritis; SLE: Systemic Lupus Erythematosus.

## Data Availability

The data and supportive information are available within the article.

## LIST OF ABBREVIATIONS

ACPAs: Anti-Citrullinated Protein Antibodies
ACR: American College of Rheumatology
ANA: Antinuclear Antibodies
anti-CCP: Anti-Cyclic Citrullinated Peptide
DAS-28: Disease Activity Score in 28 Joints
DMARDs: Disease-Modifying Anti-rheumatic Drugs
SLE: Systemic Lupus Erythematosus
dsDNA: Anti-double-stranded DNA Antibodies
ELISA: Enzyme-linked Immunosorbent Assays
EULAR: European League Against Rheumatism
FDR: False Discovery Rate
RA: Rheumatoid arthritis
RF: Rheumatoid Factor
ROS: Reactive Oxygen Species
SLEDAI: Systemic Lupus Erythematosus Disease Activity Index
VC: Vitamin C
VD: Vitamin D
VK: Vitamin K

## STANDARDS OF REPORTING

STROBE guidelines were followed.
